# Automatic dementia screening and scoring by applying deep learning on clock-drawing tests

**DOI:** 10.1038/s41598-020-74710-9

**Published:** 2020-11-30

**Authors:** Shuqing Chen, Daniel Stromer, Harb Alnasser Alabdalrahim, Stefan Schwab, Markus Weih, Andreas Maier

**Affiliations:** 1 Pattern Recognition Lab, Computer Science, Friedrich-Alexander-Universität Erlangen-Nürnberg, 91058 Erlangen, Germany; 2 Department of Neurology, Friedrich-Alexander-Universität Erlangen-Nürnberg, 91054 Erlangen, Germany

**Keywords:** Image processing, Machine learning

## Abstract

Dementia is one of the most common neurological syndromes in the world. Usually, diagnoses are made based on paper-and-pencil tests and scored depending on personal judgments of experts. This technique can introduce errors and has high inter-rater variability. To overcome these issues, we present an automatic assessment of the widely used paper-based clock-drawing test by means of deep neural networks. Our study includes a comparison of three modern architectures: VGG16, ResNet-152, and DenseNet-121. The dataset consisted of 1315 individuals. To deal with the limited amount of data, which also included several dementia types, we used optimization strategies for training the neural network. The outcome of our work is a standardized and digital estimation of the dementia screening result and severity level for an individual. We achieved accuracies of 96.65% for screening and up to 98.54% for scoring, overcoming the reported state-of-the-art as well as human accuracies. Due to the digital format, the paper-based test can be simply scanned by using a mobile device and then be evaluated also in areas where there is a staff shortage or where no clinical experts are available.

## Introduction

Dementia is one of the most common neurological syndromes in the world, in which the memory and the ability to perform daily life activities deteriorates and gets worse. According to the World Health Organization, around 50 million people currently have dementia and 10 million new cases are diagnosed each year^1^. Based on US numbers, one in 10 people of age 65 and older lives with Alzheimer’s dementia and the total number of cases are projected to more than double by 2050 from 5.8 million to 13.8 million^2^. Furthermore, around 60% of affected people live in low- and middle-income countries which sometimes have bad access to healthcare and limited money that can be spent for medical assistance or medication.

As of today, there is no cure for these kinds of syndromes, but early detection and subsequent active management can dramatically improve patients’ and caregivers’ life qualities^3–6^. Therefore, it is very important to develop an optimized diagnosing scheme, which can help to detect these syndromes as early as possible. Furthermore, the method has to be available in a more digital manner such that more people can access it from anywhere in the world.

In this work, two different terms are used for diagnosing dementia. ‘Screening’ is used to distinguish between sick and healthy individuals without differentiating between the levels of dementia or the state of mental healthiness. ‘Scoring’ implies giving scores to a performed dementia test. The score quantifies the progress of the disease and highly correlates to the patient’s mental state. Note that it includes scoring the state of both, healthy and sick individuals.

A test that is commonly used for screening and scoring dementia in the clinical routine is the so called ‘Clock-drawing test’ (CDT)^7,8^. The CDT is a brief, rapid and inexpensive paper-and-pencil cognitive screening tool also used to test for Parkinson or screening other cognitive changes^9^. Within this test, an individual has to manually complete a clock on a piece of paper by drawing the numbers 1 to 12 as well as the clock hands for a certain time setting into a preprinted empty circle. The result can then be screened and scored according to scoring systems.

Several kinds of scoring systems are commonly used, such as MiniCog, Libon, Manos, Rouleau, Royall, and Shulman^10–15^. The Shulman method^15^, which provides valuable diagnostic information, is reliable, easy, valid and widely known and accepted in Germany. During active professional training for physicians after approval of antidementive drugs like donepezil and memantine in the 90ies until 2012, when the drugs were generic available, usually all treating physicians were trained and provided with the Shulman scheme by pharmaceutical companies, since regulation required regular assessment of treatment efficacy. Therefore, we selected the Shulman scoring system to score drawn clocks in this study. However, it also comes with some downsides:

1. The system tends to remain relatively vague, leading to high inter-rater reliability since it depends on a clinician’s judgement for some generally described cases. For example, common scoring systems denote a pathological test when particular aspects of clock hands are ‘not correct’ without stating explicit criteria for wrong clock hands.
2. The test takes usually place at the clinic. The individual and family have to go through the long process of booking an appointment, waiting, and being dependent on the neurologist who is making the scoring decision, which requires efforts and patience.
3. Some bias in the clinic and judgement might take place depending on the subject’s appearance, the family, or the financial and social status.

These drawbacks show that it is necessary and required to establish an automatic method to optimize and automate the assessment of the CDT able to eliminate the bias in the scoring decision. In this work, we proposed a modern machine-learning (ML) method, in particular, a deep neural network to solve this problem. Deep learning (DL) has proven increased accuracies for traditional ML applications like classification, regression, segmentation, and others in the field of computer vision or speech recognition^16–20^. What leverages DL over classical ML techniques is that it does not require specific feature extraction but the neural networks are trained to automatically detect and recognize patterns that fit best for the given data. Recently, DL is a very prominent research field of modern healthcare, reaching also product level for some applications^21^.

Up to now, only little attention has been paid to amortization of the CDT-based screening or scoring of neurological diseases by means of deep neural networks. Harbi et al. published work on digitizing and interpretation the handwritten CDT but not screening and/or scoring^22,23^. Another work investigates the digital clock-drawing test (DCDT) in contrast to the CDT where the author stated concerns about the assessment of the manual CDT scoring systems^24^. Here, the authors did not use the usual pen-and-pencil test but a digital ballpoint pen that gives feedback on its relative position. Subsequently, software analyzes the received data by using classical ML algorithms like Support Vector Machines (SVM), random forests, and some others as classifiers for scoring.

The screening and the scoring of the CDT images can be seen as an image classification task. In the last decades, image classification achieved great success. According to the records of the common image classification challenges like MNIST^25^, ImageNet^26^, and CIFAR^27^, CNN-based methods provide better results than traditional ML-based strategies and regular fully connected deep neural networks. For image data with over hundreds of pixels, a fully connected architecture will generate millions of parameters, which is not just computationally expensive but also can lead to overfitting. This limitation can be well resolved with a convolutional neural network which analyzes features of images.

For our study, we investigated the use of the image classification neural networks for CDT evaluation. We compared Visual Geometry Group (VGG16)^28^, Residual Networks(ResNet-152)^29^, and DenseNet (DenseNet-121)^30^, which provided the most competitive results in the image classification challenges, to classify CDT images for (1) screening and (2) scoring dementia. Our solution skips the digitization steps proposed by Harbi et al. in^22,23^ by classifying the CDT directly from the hand-drawn image. To the best of our knowledge, our work is the first study to investigate and compare these successful image classification DL networks for the evaluation of CDT tests. Furthermore, we are the first who investigate the influence of the data selection on the CDT classification accuracy. After explaining the CDT and the study data in detail, we describe tested neural network architectures screening and scoring dementia including the experimental setup and parameters. In the following evaluation, we compare different neural network architectures and models and improved the performance of the image classifier by using manifold-learning based data selection for model training. In addition, we demonstrate how the proposed algorithm outperforms the performance of ML-based classifiers such as the one proposed by Souillard-Mandar et al.^24^ before discussing the outcomes and concluding the manuscript.

## Material

### Clock-drawing test and scoring system

**Figure 1:**
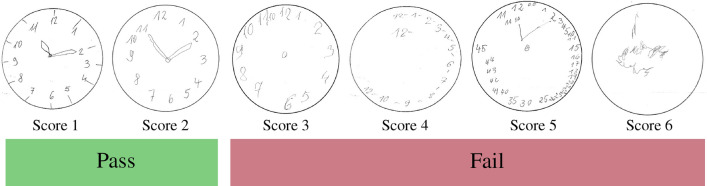
Scoring and screening of study CDTs. The CDTs are binary classified into ‘Pass’ or ‘Fail’ for dementia screening. For scoring, six classes/scores exists – ‘Score 1’ to ‘Score 6’. The images show an exemplary CDT from the study for each score where one can see the the clocks’ get worse with increasing scores.

Within a CDT, an individual is getting presented a piece of paper with a preprinted circle. The individual is then asked to draw clock numbers from 1 to 12 and clock hands that point to a specific time setting. In this study, the clinical partner (Nürnberg clinic for Neurology) uses the time setting ‘11:10 o’clock’. As illustrated in Fig. 1 (Score 1), for this command, the clock hands’ have to be located on ‘11’ and ‘2’. However in reality, the command ‘10 past 11’ can confuse individuals and make them draw one clock-hand on ‘11’ and the one on ‘10’ instead of ‘2’. The command also requires the subject to place his hands on the left and right sides of the upper quadrants of the circle, and thus, requires greater demands on executive functions by the frontal lobes of the brain. Furthermore, this setting is proved through practice to be the most sensitive setting to neurocognitive dysfunctions^7^.

According to the clock drawings produced by the subject, a score is given by adding or subtracting points depending on the level of complexity and types of the common clock drawing patterns. In this study, the Shulmann system was selected. According to Shulman et al., scores vary from 1 to 6 and increase according to the severity/status of dementia. ‘Score 1’ describes a healthy subject where no dementia or other related syndromes are present. In contrast, ‘Score 6’ indicates that the subject is totally unable to draw a reasonable clock or even anything related to a clock. To summarize the Shulmann scoring system, the ‘Score 1’ refers to the state ‘perfect’, ‘Score 2’ refers to ‘minor visuospatial errors’, ‘Score 3’ refers to ‘error in denoting the time as 11:10’, ‘Score 4’ refers to ‘moderate visuospatial errors’, ‘Score 5’ refers to severely disorganized spacing’, and ‘Score 6’ refers to ‘no reasonable attempt of a clock’. As already described in the introduction, the evaluation with this system tends to be vague with high inter-rater reliability making it hard to compare results between different clinicians or clinics. An automated diagnosis of the tests such as by means of the proposed solution will help to automatize and standardize the CDT.

**Table 1:** Subject counts for screening and scoring. The total count of subjects is 1315. Screening: The count for ‘Fail’ is larger than ‘Pass’ by about 23%. Scoring: The largest group has ‘Score 3’ while the total count of subjects is 1315.

Screening: ‘Pass’		Screening: ‘Fail’				
Score 1	Score 2	Score 3	Score 4	Score 5	Score 6	Total
240	351	445	152	92	35	1315

### Data description

#### Data collection

Data about subjects and the neuro-psychological test-results were collected from out-patients, who were referred to the general neuro-psychiatric clinic in Nürnberg, Germany, starting from July 2018. The data was collected after the approval of the local ethics committee. Subjects were of age between 18 and 98 years with a mean of $69.8\, \hbox {y} \pm 14.7$ years. The study consists of 58.1% females and 41.9% males. In total, the database consists of 1315 CDTs. The labels are generated by a professionally trained study nurse and verified by an expert and treating physician. The expert is allowed to change the label, if necessary. The changes made have been transparently documented on the clock drawing sheet. Table 1 shows the distribution of the subjects for the six scores. 591 CDTs are labeled as ‘Pass’, another 724 as ‘Fail’ (screening case). ‘Score 3’ has the highest total count and ‘Score 6’ the lowest. Figure 1 illustrates examples from the database for each score where it can be seen that the quality of the clocks decreases with increasing scores.

Subjects had no evidence of gross visual impairment (decreased ability to see to a degree that causes problems not fixable by usual means, such as glasses) and did not have any sign of aphasia (the inability or impaired ability to understand or produce speech, as a result of brain damage). However, patients of the study suffered from a wide range of diagnoses: organic brain disease, Parkinson’s disease, epilepsy, depression, subjective memory impairment, mild cognitive impairment to mild, moderate and severe dementia. Most of the patients suffered from Alzheimer’s dementia, besides to vascular, frontotemporal, or mixed dementia. Therefore, most of the time, unaffected relatives of patients gave the informed consent in the study.

All diagnoses were coded according to the ICD-10 criteria. Usually there was no attempt to use results of the clock test in the diagnostic workup. Patients received standard outpatient medical diagnostic workup and treatment. Other psychological tests beside CDT were the Mini-Mental Status Test (MMST), Demtect or the Moca-Test^31–33^. CDT was conducted and assessed as described in the previous section: After the informed consent and checking of adequate hearing and vision, patients were given the paper sheet with a drawn circle and asked to add clock numbers 1–12 and to draw the clock hands indicating ‘11:10’.

#### Data preparation and pre-processing

The drawn clock pages were digitized by a conventional scanning procedure using a commercial black and white scanner. The resulting images were 256-bit grey-scale ‘.png’ images, with 100 dpi (dot per inch), and a size of $849\,\mathrm {pixels}\times 1168\,\mathrm {pixels}$ (c.f., Fig. 1). Within the study, 1315 clock images from 1315 subjects were collected, which is comparably small to the well known data sets available online for general classification tasks like MNIST or ImageNet^25,26^. In contrast, an entire research field within DL focuses on learning from less data, especially in the field of healthcare where data is limited^34,35^. To counter the small data set, we chose to artificially enlarge it by using label-preserving transformations. In particular, we used well-known data augmentation techniques^36^: random rotations to the images in the range of $\pm 30$ degree, random re-sized cropping where we cropped images and re-sized them to $224\,\mathrm {pixels}\times 224\,\mathrm {pixels}$, and/or random horizontal flipping where an image can be randomly either flipped or kept without flipping. Furthermore, we normalized the images to mean intensity values of 0.485, 0.456, 0.406, and standard deviations of 0.229, 0.224, 0.225 (values given from the ImageNet data set). To ensure fast data processing on the GPU, all images were transformed into tensors. Furthermore we used tensor batches of different sizes in the range of 4–32 to investigate their effect on the classifiers’ accuracy.

**Figure 2:**
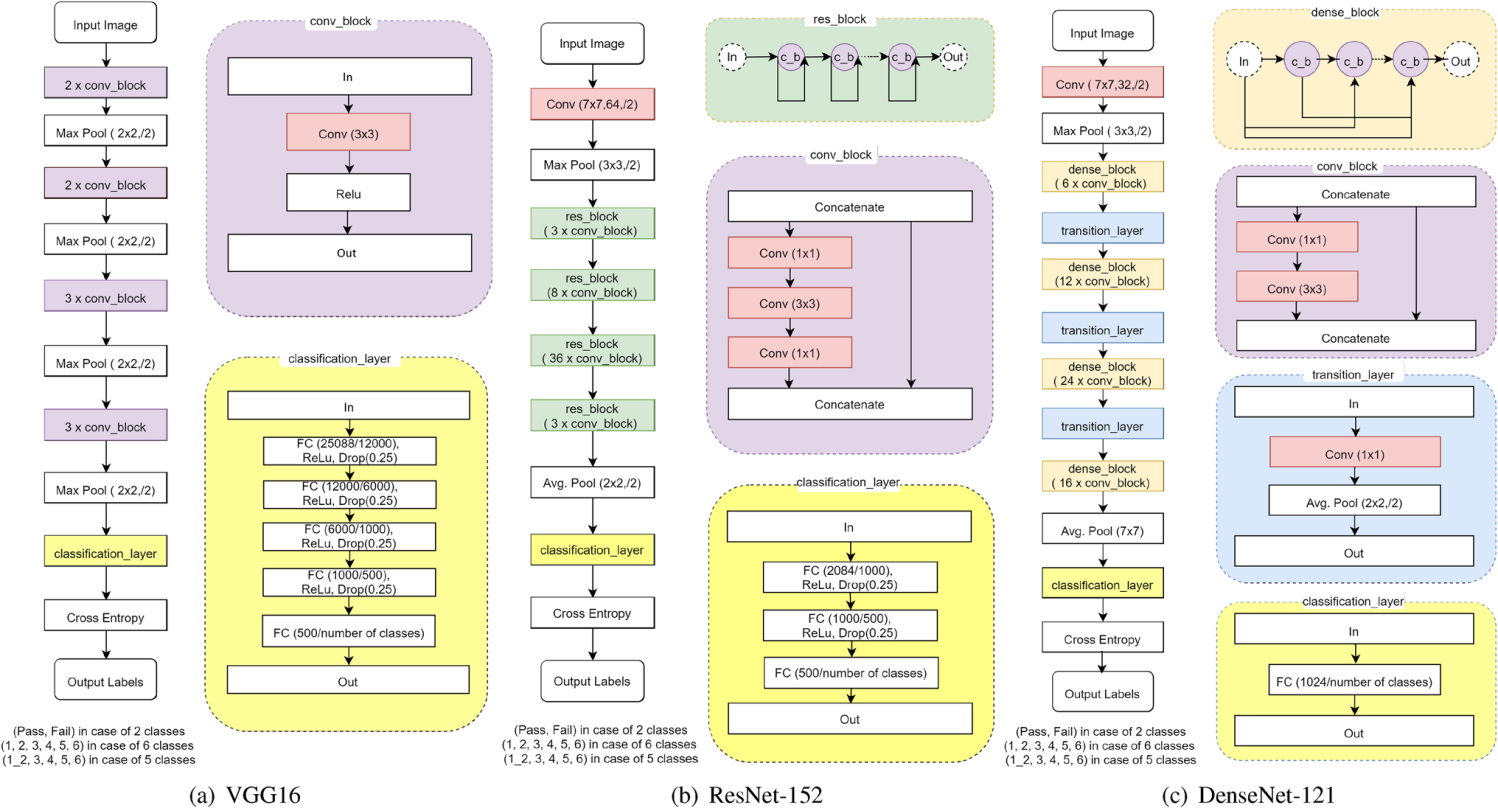
(**a**) VGG16, (**b**) ResNet-152, and (**c**) DenseNet-121 neural network architectures with modified classification layer.

## Methods

### Deep neural networks and transfer learning

Deep neural networks are DL architectures composed of multiple layers between input and output^37^. Layers consist of multitudes of neurons connected to each other where connections are assigned with numerical weights. Whereas the bottom layers contain low-dimensional features (e.g., edges/intensities), the top layers mostly store high-dimensional, class-specific information^38^. During training, inputs (here: images) are fed into the network and an output is calculated (here: ‘Score 1 to 6’ or ‘Fail/Pass’). To increase the classifiers’ accuracy and minimize a certain loss, the weights are updated using backpropagation.

Usually, these deep neural networks are rather ‘data-hungry’ meaning that a lot of training examples (several thousands or more) have to be used to generate accurate results which also comes with high training time. To compensate these missing data and decrease training time, transfer learning can be used^16,39,40^. Transfer learning leverages knowledge learned from similar tasks where a lot of labeled data is available and aims to reduce the amount of training data needed for a target classification problem. Specific models are pre-trained and then modified to fit certain use cases. Usually, predominantly bottom pre-trained layers are used whereas top-layers containing label-specific information are dropped. This strategy is very efficient for small sets of target data such as the given case^41^.

We selected three pre-trained models for our experiments: VGG16, ResNet-152, and DenseNet-121^28–30^. In their originate versions, all three network architectures were built to classify 1000 categories of images (ImageNet classes) trained on $1\,{\rm million}$ inputs achieving state-of-the-art performance. To tailor these pre-trained models to a specific target case, we chose to modify the classification layer to adapt it to our use case and describe these adaptions the following sections.

The VGG16 neural network shown in Fig. 2a has rather small convolution kernel sizes of $3\times 3$. Hereby, the combination of multiple smaller kernels emulate larger receptive fields. Furthermore, VGG16 has 16 layers with max pooling between some of them (stride: 2, size: 2)^28^. Illustrated in Fig. 2b, ResNet-152 with its up to 152 layers is known for having residual blocks instead of computing entirely new representations as done by VGG16. It also iteratively refines its input representations and allows for removal of connections (skip connections) for solving the vanishing/exploding gradient problem^29^. In contrast, the DenseNet-121 architecture shown in Fig. 2c tries to ensure maximum information flow by connecting every layer directly with each other and thus to leverage feature reuse. This significantly reduces the parameter space compared to deep nets such as ResNet-152. In addition, for all networks, the convolutional block is illustrated in the purple box of Fig. 2, the residual block for ResNet-152 in the greenish, and the dense block for DenseNet-121 in the orange.

### Manifold-learning based data selection

Usually for model training, the entire data set was randomly divided into a training set and a testing set with a splitting ratio (e.g. 80%:20%). For better generalization and to avoid over-fitting, we created a validation set for the training (20% randomly selected training images). In total, the 1315 images were split into 842 training, 210 validation, and 263 testing images. However, the random data selection can not guarantee that the classifier is learning each provided data type. The bias caused by random selection can likely result in concentrating on one class over the other, especially for a small data set. As a practical example, the algorithm can end up with overrating images for ‘Pass’ compared to ‘Fail’. Later on, when validating, it might be the case that most of the images of ‘Pass’ are in the test set. As a result, the classifier will very likely misclassify these images since it has not learned many features and examples from them. To counter this, an appropriate class distribution in training, validation, and test sets has to be determined. To take all these considerations into account, we used the method proposed by Chen et al. in^42^. First, dimensionality reduction algorithms^43^ were used to see the distribution of the entire data. Next, clustering techniques help to find the similar data. Subsequently, a random selection with previous splitting ratio within the clusters was performed. This manifold-learning based data selection can avoid the previous mentioned bias on one certain kind of data. In addition, it ensures that the neural networks can learn all possible cases during training and hence, generalize well. We employed the Matlab toolbox for dimensionality reduction^44^ for the manifold-learning based data selection.

### Screening of dementia

#### Data selection and adapted network architectures

The images were split into two main classes – ‘Pass’ (591 images) or ‘Fail’(724 images) as can be seen in Table 1. Any test that achieved a score of ‘1’ or ‘2’ was labeled as ‘Pass’, whereas tests that had higher scores were considered labeled ‘Fail’.

To tailor all three pre-trained model architectures –VGG16, ResNet-152 and DenseNet-121– to our binary class problem, the last layer (i. e., classification layer) of each architecture was replaced by an output layer categorising into 2-classes instead of 1000 (yellow boxes in Fig. 2). For VGG16 shown in Fig. 2a, we added two more fully-connected (FC) to the original two FC layers followed by a FC-softmax layer predicting the two classes. The ResNet-152 and DenseNet-121 classification layers illustrated in Fig. 2b and Fig. 2c were just adapted for the number of classes.

#### Loss function

In all our experiments, the cross-entropy (CE) loss function, also known as *log loss*, was used. The CE loss is given by

1 $$\begin{aligned} CE_{loss} = -\sum _{c=1}^{C} y_{o,c}\, \log (p_{o,c}). \end{aligned}$$

*C* denotes the number of classes, *c* the class iterator, *y* is the true class indication of *c* for an observation *o*, and *p* the predicted class score. The CE loss measures the performance of a classification model, where the output of the classifier is a probability between 0 and 1^45^. It increases when the predicted probability *p* diverges from the actual label *y*. For example, predicting a probability value of 0.025 when the actual observation label is 1 would correlate to a high CE loss value. A perfect model would have a log loss of 0, which means that the prediction probability is 1 (or 0) and the actual label is 1 (or 0), too. In the case of binary classification (screening), $C=2$ and the $CE_{loss}$ computation for an observation *o* reduces to

2 $$\begin{aligned} CE_{loss} = -\sum _{c=1}^{2} y_{o,c}\, \log (p_{o,c}) = -\left( y_{o,1}\,\log (p_{o,1}) + (1-y_{o,1})\,\log (1-p_{o,1})\right) \end{aligned}$$

#### Optimization algorithms

Optimization algorithms are used to minimize/maximize a loss function of a model. Optimization algorithms used in this work are: adaptive moment estimation (Adam), stochastic gradient descent (SGD), and root mean squared probability (RMSprop)^46–48^. The SGD algorithm updates the model variables by computing the loss function gradient sample-by-sample, instead of using the complete training set. Therefore, SGD is considered to be faster than other optimization algorithms but can also cause an unstable loss function in the updating process^47^. The Adam optimization algorithm just computes the first-order derivatives with the advantage of less memory consumption. Adam keeps the previous squared first-order derivatives as well as the past first-order derivatives of the loss function, and both the first-derivatives and their squares usually decay exponentially during training^46^. RMSprop is an optimization algorithm well-known in the world of DL that is not officially published but mentioned first in a lecture^48^. It basically adapts the learning rate by dividing an exponentially decreasing average of squared gradients. We configured learning rate values ranging from 0.0001 to 0.1 and the SGD’s momentum value was set to 0.9.

**Learning rate scheduler.** When using SGD, we usually have to provide a hyper parameter called the learning rate (LR), which is a positive number, and we have to experiment a lot to find the right value of the LR that gives the neural network its best performance. Using a large LR makes the training diverge and sometimes miss the minimum of the loss function. In contrast, a small LR makes the training converge very slowly towards the loss. To counter this, a so called learning rate scheduler (LRS) is usually proposed for solving the problem of choosing the right value. The LRS is used to change the learning rate during the training progress gradually to overcome the aforementioned issues.

In this work, we discovered that using a varying learning rate yields in better results compared to a fixed learning rate during training. We used the Step learning rate scheduler (StepLR) offered by PyTorch with a step size of 7. This kind of learning rate scheduler will decrease the learning rate from its initial value each 7 epochs by a factor $\mathrm {gamma}=0.1$. This step makes the learning rate decay slowly towards the minimum of the loss function.

#### Experimental setup

All three models were trained on the CDT image training database. For the experiments, we selected random distributions of the images within the train, validation and test sets and compared this approach to the three dimensionality reduction methods. The experimental setups are shown in detail in Appendix. The table describes the used parameters for each experiment, the modified pre-trained models used with the respective optimization algorithm, learning rate, learning rate scheduler, step size, batch size and loss function. We used PyTorch (version 1.0.1.Post2), an open-source Python-based ML library, on Google Colab (a Jupyter-based notebook cloud environment) for free usage of a NVIDIA Tesla K80 GPU (currently the available GPU is a NVIDIA Tesla T4)^49^. Transfer-learning was done by using ImageNet pre-trained models for all our experiments. All models were trained with early stopping techniques (on the plateauing of the loss function) to be able to compare results and to select the best. In particular, we investigated the influence of the data selection on the classification accuracy. We compared principal component analysis (PCA), t-Distributed Stochastic Neighbor Embedding (t-SNE), and Local Linear Embedding (LLE)^50–52^ for the manifold-learning based data selection. kNN-Clustering as performed with $k=3$. To verify the robustness of our method, a five-fold cross validation was performed. For each fold of the manifold-learning based data selection, we randomly selected data from each cluster with the same splitting ratio as the random data selection.

### Scoring of dementia

#### Data selection and adapted network architectures

For the use case of scoring, the image data set was split into six score classes with ‘Score 1’ being a perfect clock drawing (i.e., the tested subject had no detectable dementia), and ‘Score 6’ being not able to draw a reasonable clock at all (i.e., the tested subject has a very advanced case of dementia).

To extend the pre-trained networks to a 6-class prediction model, we adapted the classification layer. To estimate six instead of two classes, we implemented a one-hot vector encoding indicating the result of one of the six classes.

#### Weighted loss function

Again, CE loss was used as loss function, however in this case, $C>2$ (i.e., it is a multi-label classification task) such that we calculated the loss for each class label per observation separately and then summed the results according to Eq. (1). Another point to consider for the loss function is the available images used for conducting experiments where the classes are imbalanced and this is not the preferred case when using deep learning techniques. To counter this, we integrated the median frequency balancing technique for calculating the weight for each class^18^. We first search for ($f_{c}$): the ratio of images in each class to the total number of the images. Next, we compute the median of all of these values and finally divide it by each $f_{c}$ according to

3 $$\begin{aligned} W_{c} = \frac{\text {median}(f)}{f_{c}} \end{aligned}$$

The calculated weights are then passed to the CE loss function, respectively.

#### Experimental setup

We used a similar experimental setup as within the screening procedure including the optimizer and learning rate scheduler. The optimization algorithms we used for the multi-label classification task were Adam, SGD, and RMSprop. Learning rate values ranged from 0.0001 to 0.1 and the SGD’s momentum value was 9. Also, scoring of dementia was evaluated with five-fold cross validate strategy.

**Table 2:** Averaged binary classification accuracy results for the experiments with VGG16, ResNet-152 and DenseNet-121.

Index	Data distribution	VGG16	ResNet-152	DenseNet-121
1	randomly	0.9055	0.9405	0.9601
2	t-SNE	0.8577	0.8552	0.9521
3	PCA	0.9102	0.9422	0.9640
4	LLE	0.9359	0.9490	**0**.**9665**

## Results

### Screening of dementia

The outcomes for screening are shown in Table 2. The table shows a summary of the used nets, manifold-learning techniques, and the corresponding resulting accuracy (number of correct divided by number of wrong predictions) for the best two performing architectures is shown. Note that the accuracy has been determined by averaging over five-fold cross validation. We concentrated on the best result of each experiment type. The best accuracy is reached with DenseNet-121 and LLE reaching 96.7%. Overall, the DenseNet-121 results achieve the best results compared to ResNet-152 and VGG16. ResNet-152 also outperforms VGG16 except for t-SNE.

**Figure 3:**
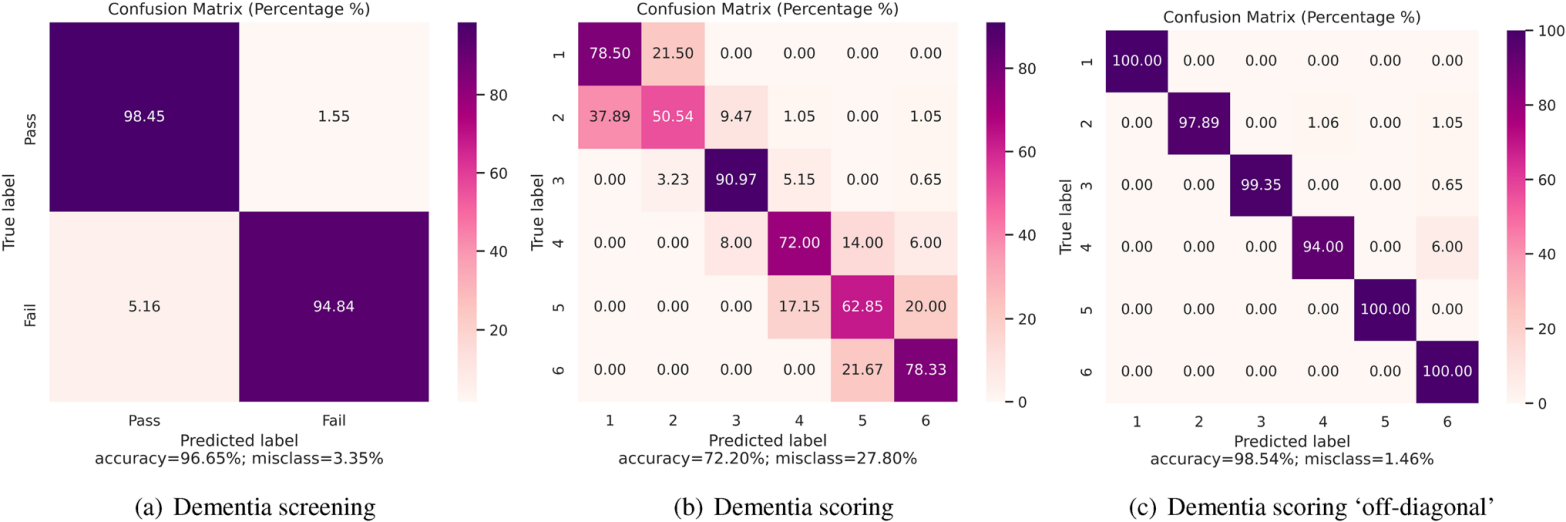
Average confusion matrix visualization of a five-fold cross validation. (**a**) The resulting confusion matrix of the best performing classifier for screening for dementia—DenseNet-121 with LLE. (**b**) The confusion matrix for scoring dementia with DenseNet-121 and LLE. (**c**) In the case where off-diagonal mislabelling is considered to be correct, the accuracy dramatically increases.

**Table 3:** Above: dementia scoring results for DenseNet-121 and LLE. Bold denotes the highest result. Below: dementia scoring with off-diagonal acceptance for DenseNet-121 and LLE. The measures show very accurate results.

Class	Precision	Recall	f1-score	AUC
Score 1	0.67	0.79	0.73	0.84
Score 2	0.67	0.51	0.58	0.72
Score 3	**0.84**	**0.91**	**0.87**	**0.93**
Score 4	0.76	0.72	0.74	0.84
Score 5	0.64	0.63	0.63	0.80
Score 6	0.74	0.78	0.76	0.88

Class	Precision	Recall	f1-score	AUC
Score 1	1.00	1.00	1.00	1.00
Score 2	1.00	0.98	0.99	0.99
Score 3	1.00	0.99	0.99	1.00
Score 4	0.99	0.94	0.96	0.97
Score 5	1.00	1.00	1.00	1.00
Score 6	0.93	1.00	0.96	0.99

When comparing the dimensionality reduction approaches, LLE outperforms all others whereas t-SNE is in every case worse than random selection. The corresponding confusion matrix of the best performing model is shown in Fig. 3a (DenseNet-121, RMSprop, LLE). When analyzing the diagonal of the confusion matrix, it can be seen that the model has a good performance. The accuracy for class ‘Pass’ achieves 98.45% where it is 94.84% for ‘Fail’. In addition, extended performance measures for the DenseNet-121 with LLE show very accurate results with precision $=0.95$, recall $=0.95$, f1-score $=0.96$, and AUC $=0.97$.

### Scoring of dementia

The screening experiments revealed that DenseNet-121 with LLE performed best. The experiments with the architectures and dimensionality reduction techniques showed similar trends as within the screening experiments. Due to the complexity of the scoring results, we concentrate on the best approach for the screening evaluation in this section.

The evaluation yielded the confusion matrix shown in Fig. 3b with an averaged accuracy of 0.722. One can see, that mostly misclassification is happening on the off-diagonal which mean s that a neighboring scores is estimated instead of the true label. Especially differentiating between ‘Score 1’ and ‘Score 2’ fails more often, however, both score belong to healthy individuals such that this has no major impact on the clinical outcome. Therefore, we allowed the images on the off-diagonal of the correlation matrix to be considered as correct. This confusion matrix is shown in Fig. 3c. The results with LLE and this allowance are very good and surpassed the literature results. The scoring results for this version are shown in Table 3. It can be concluded that the outcomes are very accurate with a f1-score always above 0.96 averaged over all six classes. On average, DenseNet-121 reached a high accuracy of 0.9854.

The corresponding confusion matrix in Fig. 3c shows that for ‘Score 1’ to ‘Score 3’, ’Score 5’ and ’Score 6’, the algorithm shows perfect scores on the data set. In 6% of the cases, ‘Score 4’ was mislabeled to be ‘Score 6’. However, in total, the accuracy is still above the non-automatically reported evaluation results.

## Discussion

The CDT scoring and classification system proposed by Shulman is generally described and can be interpreted differently from one clinician to another. Therefore, an automated screening system for distinguishing sick from healthy people and scoring those tests is highly beneficial in clinical practice. In our work, we propose an automated deep learning based technique to support clinicians in achieving more standardized outcomes compared to the traditional method of manual scoring. Automatizing these tests can help to triage individuals and relief staff and experts in their daily routine. Another advantage is that tests can be made anywhere, images can be scanned via mobile devices, and sent to evaluation centers/software anywhere in the world such that this digital solution is accessible everywhere.

To automatize the CDT, traditional ML-based approaches on digital clock-drawing test have been well described in the literature^24^. However, no fully automated processing or deep learning works have been shown until now. Our method overcomes this issues by using scanned clocks as input and just predicting the results. To make it even more digital, fully digitized scans can be made in the future, where people paint on mobile devices, such as tablets, to skip the document digitization process. If accuracies still hold for the fully digitized version in the future – and also if this is accepted by the mainly elderly population. Also the trained experts at the moment are used to evaluate paper-drawn tests and digital ones could introduce artifacts into the scoring such that we decided to go this way.

Our work achieved reliable and highly accurate results for the underlying rather small and imbalanced data set. We involved the classes’ weights into our loss calculations, and used a decreasing learning rate that could converge to the minimum loss reliably. We noticed that using a random distribution – especially in the case of a rather small data set – will result in a lower performance. Sorting the images manually is not an option as this may bias the algorithm as well as that in case of big data sets, it is just not feasible anymore. To remedy this, we recommend using a dimensionality reduction technique like PCA, t-SNE, or LLE. Those methods check the images clusters their similarity within those. We have seen that in the case of binary classification (screening) as well as multi-label classification (scoring), LLE performed best when combined with DenseNet-121 and RMSprop.

Experiments with pre-trained VGG16, DenseNet-121 and ResNet-152 show that VGG16 is giving a variety of results ranging from 85.77 to 93.59,% which is due to that VGG16 is very dependent on the underlying data set. DenseNet-121 showed a smaller range of accuracies from 95.21 to 96.65%, which means that DenseNet-121 is more independent of the data set and much more stable than VGG16. It can be due to the deeper structure of DenseNet.

We also compared the dimensionality reduction techniques PCA, t-SNE and LLE. For all of three networks, t-SNE didn’t show good results. The reason could be that, the projected points of our data formed a evenly distributed circle by performing t-SNE, which was difficult to be well clustered.

The overall performance achieved using DenseNet-121 and LLE has surpassed the reported literature results^24^ for screening with an averaged AUC of 93%. Furthermore, the method outperformed the existing clinical scoring systems, which AUCs were in the range of 66–79%, depending on the underlying data set^24^. With the off-diagonal deviation allowance of the correlation matrix, the classification achieved very high AUCs.

It should be mentioned that after seeing many subject clocks, there are still test results that are hard to assess even by professionals. This uncertainties of course also influence the results of an automatic algorithm. An entire research field within deep learning is working on this field of uncertainty measures^53,54^. Including this into our work was out of scope but is worth investigating in the future. Also averaging over ratings of more experts and applying of further optimization techniques may remedy this problem^55,56^. Especially interesting would be reports and clocks from different populations, rated by clinicians from different hospitals/continents, but such data is hard to get for researches if they are not published open source. When it comes to product level, hospital-individualized training would be the better option for training those neural nets – at least to this point. In addition, different clock times (here: 11:10) can lead to different results.

Generally, there exist several schemes and strategies for detection of Alzheimer disease, i.e. image, speech and handwritten based methods. Thanks to the high detection accuracy, the detection with MRI brain image is the most often used method. However, MRI image capture is expansive and time consuming. It can not be repeated frequently for a patient follow up study. On the other hand, the speech based method is convenient for both patient and doctor and can be made periodically. Though, the performance speech method relies highly on the language. To achieve a high recognition accuracy, transfer learning shall be applied to adapt the network on certain language. A cross-lingual detection is very challenge and kept as an open issue. Contrast to these two methods, Clock test from category handwritten based method has such advantages as easy to apply, lingual independent, and high detection accuracy.

The classifier we built is a convenient and low cost tool based on DL methods which can be used in hospital, practice or for nursing or home care applications with an acceptable error. In comparison to the traditional assessment schemes, our automatic screening and scoring of clock drawings shows the advantage of being independent from trained personal’s decision. Using a method like our proposed, physicians or neuro-psychologists can spent more time to focus on other routine or research work. Moreover, the automated system will prevent any possible biasing to any subject’s performance and appearance. Also, the DL classifier could be like a second opinion to the trained personal. The method can support health-care professionals to keep track of their patient’s mental status in a way more efficient way, and therefore it can be of a preventive use so that doctors can be aware when patients mental state is starting to get worse and to counteract fast.

## Conclusion

Machine-learning techniques just started to proof to be very powerful in the domain of health-care and assisting professionals in clinical decision making procedures. In this work we showed that deep learning can be a great tool to enable automatized screening and scoring for standardized neurological tests such as the clock-drawing test. Our proposed neural network achieved very high AUC and clearly outperformed reported clinical screening results by 24–27%, other machine-learning screening techniques by 4–24%, and machine-learning scoring approaches by 10–27%. The algorithm can be easily integrated into hospitals’ environments and care facilities to help monitor the state of patients with dementia. Furthermore, due to digitization, the tests can be screened and scored anywhere at any time making it accessible for everyone.

## Statement

All experiments and methods were performed in accordance with relevant guidelines and regulations. All experimental protocols were approved by a named institutional/licensing committee. The collection and usage of the data as well as experiments related to this study were approved by the local ethics committee (*Ethik-Kommission der Friedrich-Alexander-Universität Erlangen-Nürnberg*, www.ethikkommission.fau.de, $25^{\mathrm{th}}$ July 2018, 182_18 B) and all methods were carried out in accordance with their relevant guidelines and regulations. Informed consent was obtained from all subjects. In the case of patients with severe dementia, an informed consent for study participation has been obtained from legal guardians. The datasets generated during and/or analysed during the current study are available from the corresponding author on reasonable request.

## Appendix

The three pre-trained models DenseNet-121, ResNet-152, and VGG16 (columns) and the used parameters for training (rows). The last column indicates the chosen data distribution.

**Table:**

Index	Used parameters	DenseNet-121	ResNet-152	VGG16	Data distribution
1	Optimization algorithm	RMSprop	Adam	SGD	randomly
	Learning rate	0.0001	0.0005	0.001	
	Learning rate scheduler	StepLR	StepLR	StepLR	
	Step size in learning rate scheduler	7	7	7	
	Batch size	16	16	4	
	Loss function	Cross entropy	Cross entropy	Cross entropy	
2	Optimization algorithm	RMSprop	Adam	SGD	t-SNE
	Learning rate	0.0001	0.0005	0.001	
	Learning rate scheduler	StepLR	StepLR	StepLR	
	Step size in learning rate scheduler	7	7	7	
	Batch size	16	16	4	
	Loss function	Cross entropy	Cross entropy	Cross entropy	
3	Optimization algorithm	RMSprop	Adam	SGD	PCA
	Learning rate	0.0001	0.0005	0.001	
	Learning rate scheduler	StepLR	StepLR	StepLR	
	Step size in learning rate scheduler	7	7	7	
	Batch size	16	16	4	
	Loss function	Cross entropy	Cross entropy	Cross entropy	
4	Optimization algorithm	RMSprop	Adam	SGD	LLE
	Learning rate	0.0001	0.0005	0.001	
	Learning rate scheduler	StepLR	StepLR	StepLR	
	Step size in learning rate scheduler	7	7	7	
	Batch size	16	16	4	
	Loss function	Cross entropy	Cross entropy	Cross entropy	
